# Faith-Based Institutions as Venues for Obesity Prevention

**DOI:** 10.1007/s13679-017-0257-8

**Published:** 2017-04-11

**Authors:** Maria J. Maynard

**Affiliations:** School of Clinical & Applied Sciences, Leeds Beckett University, City Campus, CL 413, Calverley Building, Leeds, LS1 3HE UK

**Keywords:** Obesity, Weight loss, Faith-based health, Community, Ethnicity, Prevention

## Abstract

**Purpose of Review:**

The aim of this current narrative review is to critique the scope and value of recent studies with a focus on obesity-related health promotion in faith organizations.

**Recent Findings:**

Electronic database searches, scanning of the reference lists of identified articles, and hand searching of journals for articles written in English and published in 2013–2016 revealed 16 studies. Half of the studies involved African-Americans, in churches and with predominantly female participants. Research among other ethnic groups was more likely to be exploratory. All of the 11 studies reporting the impact of programmes on weight-related measures showed favourable outcomes. However, due to study limitations (small sample size, short duration, attrition), significant unbiased effects cannot yet be concluded for most of the interventions reviewed. Study strengths included application of theory in community engagement and detailed description of cultural tailoring.

**Summary:**

Faith organizations show promise as settings for obesity prevention among high-risk groups, particularly African-Americans. Support for progressing formative work to adequately powered, randomized controlled trials is vital. Wider involvement of diverse faith settings and targeting obesity in men and childhood would be valuable developments.

## Introduction

The goal of the World Health Organization (WHO) Action Plan 2013–2020 is to reduce the preventable and avoidable burden of morbidity, mortality, and disability due to non-communicable diseases; this includes stemming the seemingly intractable rise in the prevalence of obesity and its consequences [1]. Current predictions for reaching these goals remain bleak with global obesity prevalence set to reach 18% in men and more than 21% in women [2]. In addition to upstream policy action, support for local settings and community leaders to promote healthy food and physical activity (PA) environments and behaviours is seen as key in the prevention of obesity and related diseases [3]. Community-designed and community-delivered interventions, with culturally relevant materials, have been found to be most acceptable and most significantly influenced programme success across a range of health outcomes [4]. ‘Lay’ health workers from target communities engaged with the research can act as intermediaries between the community and research team [5, 6] and can provide credibility, expertise, and empathy in the delivery of an intervention [4]. Faith institutions are community loci which have long been involved in advocacy for social change, including health inequalities [7]. Although partnerships with the health care sector can be complex and contentious, faith and medical organizations have intersected successfully to deliver health-related programmes for a wide range of health outcomes [8]. In the WHO global strategy for diet and PA, it is suggested that these behaviours can be particularly influenced by religious institutions and recommended exploring such settings as venues for obesity prevention [9].

Ethnicity is a consistent correlate for overweight and obese children and adults, with a number of minority ethnic groups at greater risk of obesity and its consequences, compared to majority populations, partly explained by socio-economic factors [10–15]. Prevention activities have had poor reach for these groups to date and specific interventions are required to address this inequity [16]. Although there has been an overall decline in religious practice in developed Western nations [17], the majority of Americans (77% of all adults) identify with a religious faith [18]. Regular attendance at a place of worship is common among some of the ethnic groups most at risk of obesity such as Black Africans and South Asians [17, 19]. For non-denominational or new churches, which attract mainly African-American and Black migrant ethnic groups, membership increases year on year [18, 20]. Religious affiliation and attendance are also important in many low-income settings [21]. Programmes involving faith organizations may thus plausibly contribute to addressing obesity inequality among high-risk groups.

So, to what extent has the potential for faith settings in obesity prevention been realized? There has been success in effective church-based obesity interventions among African-Americans [22]. Of 18 studies targeting weight loss published 1992–2012, significant improvements in outcome were reported in 12 (70%), although in only two of the five randomized control trials (RCTs) [22]. The aim of this current narrative review is to summarize and critique the latest research on approaches to faith-based obesity prevention, how effective these interventions have proved to date, and observations on future directions.

## Obesity Prevention in Faith Organizations, 2013–2016

Primary research studies involving places of worship and weight-related outcomes, written in English and published 2013–16, were sought. Electronic searches of Medline, Science Citation Index, and PubMeD databases, scanning of the reference lists of identified articles, and hand searching of journals were conducted. No restrictions relating to participant age, ethnic group, or religion were imposed. The 16 studies in 15 articles identified reported on or proposed formative research, quasi-experimental, and cluster randomized trials that explored or tested the feasibility and efficacy of faith settings for achieving weight-related change. The focus was obesity per se for some studies, but in others, weight loss was the route to addressing risk of diabetes or cardio-vascular disease. Half of the studies were aimed at African-American men and women [23•, 24–29], with the remainder among Latinos [30, 31], South Asians [32•, 33], American Indians [34], Black Caribbeans [33], Black Africans [33, 35], Samoans [36], and general church attendees without a particular ethnic focus [37]. Studies were located in the USA [23•, 24–31, 32•, 34, 36], UK [33, 37], and Australia [35]. The two studies reporting the very earliest qualitative exploration of the potential for intervention development are not critiqued further [35, 36]. Of the substantive interventions with weight-related outcomes (*n* = 11 studies), four were faith placed (located in faith organizations without any spiritual message) [24, 29, 31, 33] and the remainder had tenets of religion integrated into the programmes (faith-based). Interventions were principally delivered to groups, and in most of the articles, the intervention sessions and cultural tailoring were richly described. The majority of studies targeted overweight participants and also included those already obese. Generally, studies were often also open to non-overweight participants, and some additionally involved the wider congregation in healthy lifestyle activities, regardless of weight status. Therefore, the studies included can be deemed as contributing to primary and secondary prevention, as these categories are often blurred in addressing obesity, as in other multi-factorial conditions [38]. In all but one study [37] (diet only) both dietary and PA behaviours were targeted to effect weight change. Intervention sessions led by members of the community trained to deliver the programme were a common feature of most of the studies. Where this was not the case, the intervention was delivered by dietitians and fitness experts [27] or a church minister who was also a trained nurse [25]. Two studies included children [33, 34] and two targeted females only [25, 34], and male participation was low (12–29% of the sample) in those studies involving men and women. The studies and their findings are further described then critiqued subsequently.

### Studies Including African Americans

A completed cluster randomized controlled trial [23•] and the protocol for a cluster RCT [28] were identified. Fit Body and Soul (FBAS) included 604 African-Americans in 20 churches [23•]. The study was a community adaptation of the US diabetes prevention program (DPP), a multi-centre clinic-based programme successful in achieving weight loss through intensive dietary and PA modification to reduce risk of type 2 diabetes [39]. Participating churches were randomized to receive FBAS or a generic health education (HE) intervention. Intervention dose was 12 1-h weekly sessions with six monthly 1-h ‘booster’ sessions. HE sessions were consistent in intervention dose and attention but involved only a prayer and discussion of a health topic. All participants completed the study, and at 3-month and 12-month follow-up, FBAS participants had significantly greater weight loss compared to the HE arm. After the success of the pilot phase [40], the wholeness, oneness, righteousness, deliverance trial (the WORD), was established, with a target sample of 450 men and women in 30 churches, and is currently in the recruitment phase [28]. The intervention will have two arms; churches will be randomized into a 16-session weight loss intervention or the same intervention followed by a 12-week weight maintenance protocol. Follow-up at 6, 12, and 18 months is planned. Comprehensive description of the session content includes detailed explanation of the spiritual message associated with each session.

One small-scale RCT and five studies with quasi-experimental designs were also conducted among African-Americans [24–26, 29]. Follow-up periods ranged from 2 to 6 months. The Living Well by Faith (LWBF) pilot programme [27] involved 106 participants in five churches randomly assigned to receive LWBF or a limited intervention control of one educational workshop. The LWBF consisted of an intensive 8-week course of bi-weekly 90-min sessions, together with individualized ‘wellness’ plans. Follow-up was 2 months. The study was completed by 97% of the LWBF group and 75% of controls. Significant decreases in weight, BMI, and body fat were evident in the intervention group compared to the controls. Seventy participants in two churches took part in the waitlist-controlled Heart Smart Church (HSC) study [24], with no loss to follow-up reported. The bespoke intervention was delivered in 3–12 group sessions, with additional individual face-to-face meetings [24].

In the remaining four studies among African-Americans, there was no control group, a pre-/post-test design was employed, and follow-up ranged from 3 to 6 months. Whisenant and colleagues [25] describe two studies. In the first of these, 35 female participants took part in a 3-week intervention designed by the researchers; 11 individuals were lost to follow-up [25]. The second study was delivered by a trained nurse congregant to 21 participants (15 female) from two churches over 12 weeks [25]. The Turn the Beat Around (TBA) study, based on the existing With Every Heartbeat is Life intervention components, was delivered in six weekly sessions to 201 men and women in nine churches, 94% of whom completed the study [26]. Feasibility of achieving weight loss using a further modification of an intervention developed from the DPP was tested in the Lifelong Prevention Program (LPP) among 13 participants, 11 of whom completed the study [29]. Sixteen group sessions were supplemented with six personalized telephone support sessions [29]. Positive weight/waist circumference outcomes were reported for all four of these quasi-experimental studies, but did not reach statistical significance in the TBA study [26].

### Other Ethnic Groups

Studies involving ethnic groups other than, or in addition to, African-Americans included one small cluster RCT [31], three pilot-scale studies with a quasi-experimental design [30, 32•, 34], and studies describing completed or proposed formative work [33, 37]. The *Un Estilo De Vida Saludable* (EVS; ‘healthy lifestyle’) study among 58 Mexican-Americans (78% female) was also an adapted version of the DPP, involving the randomization of two churches to receive either the EVS intervention or an attention-control intervention [31]. EVS was a 5-month-long programme with an intensive phase of eight 2-h weekly sessions, followed by a maintenance phase of three monthly 1-h sessions. The attention control arm received the same intervention dose and attention, in sessions providing general information on health conditions (but not diabetes) delivered by a bi-lingual nurse. In the study, overall attrition was high, with only 57% (*n* = 33) of the total sample completing the study and particularly marked in the control group (40% attrition in this group). Greater weight loss, lower waist circumference, and BMI were reported for the EVS vs. the control group.

The two studies with control groups but non-randomized design included the first pilot community-based diabetes prevention programme among Sikhs in New York [32•], and a study initiated with formative qualitative research, building to a pilot cardio-vascular disease prevention trial among Lumbee Indian women in NC, USA [34]. Sikh participants recruited in Gurdwaras (Sikh temples) were allocated to an intervention arm or control according to residence in two locations. A total of 76 participants were allocated to intervention and 50 to control. Follow-up was at 3 months and 6 months. Sessions included interactive 2-h sessions held at 3-week intervals over the 6-month study. Intervention participants also received follow-up phone calls between sessions for individualized action plans. Control participants were advised to maintain standard care and prevention activities, but received the full intervention at the end of the 6-month study period. Seventeen (22%) of the intervention participants were lost to follow-up (compared to only one in the control arm), but demographic factors were similar to those who remained in the study. In the treatment arm, weight was significantly lower than at baseline, with almost 5 lbs lost on average, although there was no significant difference in weight loss between the treatment and control groups. Formative research among the Lumbee women [34], including review of the literature, meetings with church leaders, and an assessment of potential church venues, followed by a series of stakeholder focus groups, led to the development of intervention content and delivery. Four churches were subsequently recruited and two each assigned to the intervention arm or a delayed intervention control, according to geographic location. The bespoke intervention of weekly classes over a 4-month period was delivered in all four churches; however, of the 165 participants enrolled, only 27% attended the intervention. Barriers to implementation included difficulties scheduling sessions due to competing church activities and resistance to dietary change. The impact on BMI is yet to be reported.

Also in the USA, Gutierrez et al. [30] targeted low-income, urban African-Americans, and Spanish-speaking Latino parishioners in the Fine, Fit, and Fabulous study. In this pre-/post-test intervention, 253 participants were recruited into the study of which 183 (72%) completed the programme and 63% provided sufficient data to be included in weight loss analyses. The intervention was developed by church members and consisted of a 12-week programme. Originally designed for African-Americans, the curriculum was additionally culturally and linguistically adapted for Latinos in Spanish-speaking churches; however, the article lacked detail on intervention development and content. On average, participants lost a significant 2% (4.38 lbs) of their body weight compared to baseline, with weight loss being greater for the African-Americans than that for the Latinos. Analysis stratified by BMI category indicated that percentage weight loss was greatest among those who were initially overweight (BMI 25–29 kg m^−2^) compared to those in normal or obese categories.

Feasibility of childhood obesity intervention components and delivery among children aged 8–13 years was explored in temples, mosques, and churches compared to that in schools in London, UK [33]. Potential intervention components were informed by focus groups with children and parents [41] and extant literature and tested among 155 boys and girls in schools and 33 children in places of worship in one-off sessions. Mixed qualitative and quantitative evaluation of recruitment and acceptability and feasibility of the interventions indicated straightforward recruitment of schools, multi-strategy approaches to recruitment of places of worship, low response rates of organizations, but high participation rates of individuals. Evaluation coverage, however, was more consistent in places of worship than in schools. Also in England, Lycett et al. [37] propose a small Christian church-based feasibility study among ten participants with the aim of using the findings to design a cluster RCT. No particular social or ethnic group is targeted and the intervention will be open to those who do not ordinarily attend church. The ten planned 90-min weekly sessions will focus on ‘intuitive eating’ without explicit mention of weight loss. There is no indication given that there was input by potential stakeholders in the intervention design and was the only study reviewed which did not include a PA component.

## Discussion of the Studies

### Study Design

Cluster trials are an appropriate choice for obesity prevention in faith settings, with randomization of the clusters into intervention and control arms being the most robust design [42]. Others have commented on the lack of appeal of traditional, no-intervention control arms in community settings which may hamper effective collaboration [43]. Different versions of the main intervention and other less intensive or not specifically tailored interventions as control were alternatives used. Correlation between observations on participants in the same cluster (which decreases the effective sample size) and the required adjustment for valid analysis (reducing statistical power) are some of the design and analysis challenges inherent in cluster RCTs [42]. A cluster-RCT design was used in a completed trial and in the proposal for a definitive trial, both among African-Americans, with sample sizes of 604 in 20 churches and 450 participants in 30 churches, respectively. In these studies, attention to methodological quality was evident, including favourable distribution of participants in large numbers of clusters, and at least 12-months follow-up. Power calculations were provided and clustering taken account of in sample size calculations and in statistical analyses, with details of the methods given. The Fit Body and Soul intervention group had significantly greater weight loss than the controls and attrition was <10% [23•]. Together with past studies [22], this indicates that well-designed, adequately powered obesity prevention studies among African-Americans can be conducted in faith settings and can be effective. The pragmatic selection of churches and participants means a potential lack of representativeness, and therefore, generalizability of the study findings to other settings and populations may be limited.

Studies implemented on a small scale were variously defined as pilot or feasibility, as is common in the intervention literature [44] or were not defined as such although their small sample size and other methodological limitations preclude them being viewed as definitive trials. Two small cluster RCTs were conducted with 106 African-Americans and 58 US Mexicans. Even in the small study among African-Americans, there were at least two clusters in each arm and clustering was taken into account in the statistical analyses, although the method was not provided. By contrast, the one cluster per arm in the study among Mexicans means the intervention effect is confounded by the cluster effect. Both are unlikely to be useful in determining sample size for a main trial due to the small number of clusters and participants [45]. The remaining trials were quasi-experimental studies. Studies with control groups but in which clusters were not randomized to the different study arms, ranged in sample size (*n* = 70–165) and involved two or four clusters. Comparison arms included waitlist and attention control groups. Those studies which lacked a control arm, instead utilizing pre-/post-comparison analysis, included three very small studies (*n* = 11–35) with one or two clusters; and two larger studies (*n* > 200) with 9 and 15 clusters. All of the ten pilot/feasibility studies reporting their weight-related outcomes had favourable findings; however, estimates of effects were not all significant and are likely to be biased. Small sample sizes and/or convenience sampling and attrition (ranging from 6 to 37%) in eight of these ten trials means that they are problematic with regard to the internal and external validity of study findings. A short follow-up of 6 months or less for the majority of studies reviewed does not allow the demonstration of potential sustainability of any beneficial effects; however, a long follow-up adds considerably to study costs [44]. The strength of such studies, however, is that together with formative studies, they can provide detailed information on the development, acceptability, and feasibility of the content and delivery of interventions before an investment is made in wider-scale piloting and implementation [46]. Maximizing the value of such studies requires detailed process evaluation, reported to a greater or lesser extent in the studies reviewed (see below) and commitment from journals to publish such material [47].

### Process Evaluation

Process evaluation aids assessment of the fidelity of intervention delivery, reach and dose, and can be used to document what helps and what hinders intervention delivery, including participants’ perspectives [48]. Lack of, or limited, process evaluation was a feature of the quasi-experimental studies and, surprisingly, some of the formative, developmental, or feasibility studies [24–26, 29, 30, 34]. Where process evaluation was undertaken or proposed [23•, 27, 28, 31–33, 37], methods included qualitative and quantitative approaches. In completed studies, findings were only presented in the reviewed article (or referenced in another article from the same study) for some of the studies [27, 31–33]. In complex interventions, it is difficult to tease out which individual component (e.g. diet, PA, social support) or their interactions are the most efficacious. Process evaluation can aid elucidation of underlying mechanisms of change, however, weaknesses in measures (e.g. subjective PA measures; non-validated dietary assessment tools) hinder this.

### Theoretical Frameworks

Interventions aimed at changing health behaviours can often be under-theorized [49] and in the current review there was no explicit reference to theory underpinning intervention content and delivery in some studies [25, 27, 30–31, 32•, 34], which may reflect a lack of theory development for culturally tailored interventions. The theory emphasizing individual level behaviour change can often be the focus; however, where theory was applied, the predominance of models which incorporate shaping of intentions by wider social, cultural, and environmental influences (such as social cognitive [50], Health Self-Empowerment [51], social networks, and social support theories [52]) was apparent [24, 28, 31].

Theory-driven strategies can also underpin the engagement and fostering of trust needed for effective and sustainable intervention, requiring considerable goodwill of researchers, institutions, and communities [53]. Community-based participatory research (CBPR), components of CBPR, or similar approaches to community engagement and empowerment were the most common theories applied to forging relationships between research teams and faith organizations in the studies reviewed [23•, 24, 27, 28, 31, 35, 36]. Nine core principles for CBPR have been suggested ranging from acknowledging community as the unit of identity to commitment to sustainability [54]. CBPR promises to be advantageous in navigating the challenges of obesity prevention within faith organizations. General guidance [53] as well as a full description of methods and lessons learned will aid future development of CBPR in this work. For example, Yeary and colleagues [28] describe how they collaboratively identified weight loss as a community issue and developed the overall study design, intervention components, delivery, evaluation, and dissemination, mapped to the nine core principles of CBPR. The majority of the studies involved community health workers in intervention delivery. Length of training of community health workers (CHWs) varied and details of the training received was not always explicitly described, although there were good examples of clearly explained rigorous training (see, for example, Islam et al. [32•]). It is difficult to ascertain whether the use of CHWs improves efficacy, but it is likely that such ‘community champions’ [6]; (p.96) will be important in sustainability of programmes, and ensuring community perspectives are at the centre of intervention programmes.

## Future Research Needs

Future research to expand knowledge on faith organizations as settings for obesity prevention warrants the further testing of components and adequately powered pilot and definitive trials. Robust study design and analytical techniques are required at all stages of intervention development and testing. Explicit adherence to guidelines for reporting trials, such as the Consolidated Standards of Reporting Trials (CONSORT) statement which has been extended for cluster randomized trials [55], will improve the consistency of this emerging evidence base. The studies conducted in ethnic groups other than African-Americans indicate the beginning of a broadening reach of this area of research. However, the likely efficacy of faith settings for addressing obesity among children, men, and diverse ethnic groups in faith organizations other than churches (e.g. mosques and temples) and in low-/middle-income countries represents significant gaps in the current literature. As the evidence base strengthens, it will be increasingly important to examine the appropriateness of theory application and the extent to which theory improves potential for programmes to effect change [56].

## Conclusions

Faith organizations are neighbourhood focal points and attendance at places of worship remains important for a range of communities, including those at high risk of obesity. Current research suggests continued promise in effective and sustainable obesity prevention through faith organizations among African-Americans. Additional large-scale, randomized controlled trials in this group are essential, with methodologically robust design, conduct, and analysis, reflected in the reporting. Faith-based activities are also beginning to be used to engage other high-risk populations and may emerge as significant approaches for tackling obesity inequalities in the future. Nascent evidence, however, is dominated by small-scale studies with design limitations and research in the formative and exploratory stages. Advancement of obesity prevention in faith organizations will require commitment to the timely progression of developmental work to feasibility and pilot trials in a wide range of places of worship and populations, most profitably employing theoretically underpinned community-based participatory approaches.
